# Co-production in HIV research: a case study from the COVID-19 pandemic

**DOI:** 10.1186/s40900-025-00713-3

**Published:** 2025-05-09

**Authors:** Vasiliki Papageorgiou, Jane Bruton, Halle Johnson, Silvia Petretti, Wezi Thamm, Joshua Anderson, Husseina Hamza, Helen Ward

**Affiliations:** 1 Patient Experience Research Centre, Imperial College London, London, UK; 2 Positively UK, London, UK

**Keywords:** Community-Based participatory research, COVID-19, HIV, Qualitative research, Co-Production

## Abstract

**Background:**

Co-produced research is an approach where people with lived experience voluntarily engage as collaborators throughout the entire research process. In this study, academic researchers aimed to recruit and train people living with HIV, in partnership with a community-based organisation (Positively UK), with the objective of enhancing research skills and capabilities of the HIV community to lead and/or facilitate forthcoming research initiatives and projects.

**Main body:**

Our collaborative endeavour involved a group of academic researchers, a public involvement practitioner and people living with HIV (comprising two co-researchers and three advisory group members) to design and conduct a participatory, qualitative enquiry from January to September 2021. Our study aimed to explore the experiences of people living with HIV within the United Kingdom during the COVID-19 pandemic. All co-researchers underwent rigorous and essential research training that encompassed ethics, conducting qualitative interviews, analysing transcripts and considerations pertaining to emotional well-being. Communication was conducted exclusively by phone or online throughout the project. The unpredictability associated with the pandemic necessitated an adaptive and flexible approach that encompassed personal circumstances and the intricacies of conducting research during a global health crisis. Based on our reflections, we map out our collaborative experiences and provide recommendations for the advancement of future co-produced research endeavours involving people living with HIV at each stage of the research cycle. We identify four areas of focus: (1) building relationships inclusive of trust and rapport and managing stakeholders’ expectations; (2) reciprocal learning and ensuring the amplification of all voices; (3) the necessity for flexibility and the integration of continuous reflection; and (4) the pursuit of impact that transcends traditional academic outputs.

**Conclusion:**

Participatory research and co-production are increasingly common approaches to research. These approaches have impacts on all stakeholders involved including co-researchers, public involvement practitioners, academic researchers and the wider community. In the context of this study, we demonstrate that undertaking co-production remotely and building meaningful partnerships remains possible where teams build good rapport challenging traditional power structures. The team members affirmative experiences could potentially positively affect their future participation in research, whether as participants and/or as co-researchers, as well as informing the future research strategies and decisions of researcher investigators.

## Abstract (plain language summary)

Co-production is when people with lived experience are involved in research in an equal way with researchers. In this co-produced project, we worked collaboratively as a university and charity to train a group of people living with HIV to carry out and analyse interviews in research. Our study looked at the experiences of the COVID-19 (coronavirus) pandemic among people living with HIV in the UK. Experiences were captured through interviewing people either online or by phone between January and September 2021. Conducting a co-produced research project with people living with HIV during a pandemic presented unique and unexpected challenges, which required transparency and continuously adapting to the situation. In this paper, we reflect on what we learned by working together and share recommendations for future co-produced HIV research.

We found four areas which are important:

(1) building relationships and managing expectations,

(2) joint learning and ensuring that everyone is heard,

(3) being flexible and constantly reflecting,

(4) having an impact outside of the academic world.

We know that participatory and co-production approaches are becoming more popular forms of research. They give greater possibility to benefit the people involved and the wider community. We found that it is still possible to build meaningful relationships when running a co-production project remotely. We also think that deciding to take part in future research as a participant and/or co-researcher may be influenced by having a positive experience in a co-produced research study.

## Background

Participatory research is an approach where key stakeholders (e.g. service users, carers etc.) work in partnership with researchers to design and conduct a study [1]. Co-production (or co-research) is an example of a participatory approach in which people with lived experience are integral partners throughout the research process [2]. This approach is underpinned by the principles of reciprocity; sharing of power; building and maintaining relationships; including all perspectives and skills; and respecting and valuing the knowledge of all those working together [2]. Community-based participatory research (CBPR) acknowledges how the research is led “by communities for communities” and therefore has a role of translating research findings into actionable outcomes, including social change; CBPR rests on the pillars of ethics and community empowerment [3].

Several theories may be used in co-design, co-creation, and co-production of public health research [4]. Empowerment Theory focuses on how stakeholders can be empowered in processes such as co-creation, thereby linking individual well-being to the wider sociopolitical environment [4, 5]. Additionally, the EMERGES framework (**E**nablers and Empowerment; **M**otivation to Integrate; **E**mpathy of the self and others; **R**ecovery model and medical model; **G**rowth and Transformation; **E**xclusion (Stigma and Discrimination); **S**urvivor roots/Disability roots) was developed to outline the multifaceted identities of researchers or providers with lived experience [6]. The framework was developed through a systematic review in the context of mental health [6]. Nonetheless, components of these concepts can be transferred and used in HIV research. Specifically, the experiences of exclusion, stigma, and discrimination, aligned with the potential for public involvement aimed at fostering metaphorical personal and collective growth and transformation (for example, the development of a new professional identity or seeing teammates ‘blossom’), as well as a nuanced understanding and empathy of oneself and others [6, 7].

Participatory approaches are well established in the HIV response. A prevalent slogan among people living with HIV is *“nothing about us*,* without us”* which has its origins in the in disability rights movement [8]. Community-based organisations (CBOs) have championed the Greater and Meaningful Involvement of People Living with HIV (GIPA/MIPA) since the early days of the HIV epidemic [9]. Consequently, in HIV research, participatory approaches are progressively becoming more common [10]. However, researchers have argued for clarification regarding the terminology and definitions employed, in addition to a comprehensive framework delineating roles throughout the research cycle that aligns with specific needs and capacities of stakeholders [10].

The COVID-19 pandemic resulted in health services globally, including HIV clinics, having to rapidly adapt service delivery. For HIV clinics specifically, a need to maintain HIV care was identified early on during the pandemic [11]. For example, ensuring people living with HIV had access to antiretroviral therapy; the provision of pre- and post-exposure prophylaxis and HIV prevention tools; and adapting services including routine appointments and peer support to be delivered virtually rather than in-person [11, 12]. This presented several challenges relating to digital exclusion but also concerns for privacy and confidentiality [13, 14]. Concerns were also raised about the mental health and isolation of people living with HIV, particularly as the prevalence of mental health conditions is already high in the population [13, 14]. In the United Kingdom, some clinics had HIV staff redeployed to intensive care units during COVID-19 and at the start of the pandemic, information relating to shielding advice for people living with HIV was conflicting [12, 15, 16]. Specifically, the UK government had provided conflicting advice about whether people living with HIV should shield with some people receiving letters and text messages, despite the British HIV Association (BHIVA) and the Terrence Higgins Trust confirming this was unnecessary unless they are at the highest clinical risk of severe COVID-19 (e.g. people with CD4 count < 50 cells/microL) [17–19]. Finally, there were concerns of the impacts of COVID-19 on wider determinants of health including finances, housing and employment, particularly for the most vulnerable people living with HIV [12].

The COVID-19 pandemic also introduced a unique challenge to co-producing research as government restrictions necessitated a shift to remote activities. Despite these challenges, initial insights indicated that the public were keen to continue contributing their time and expertise to support the ongoing response [20].

Our aims were to:

1. recruit, appoint and train two qualitative researchers with lived experience to conduct and analyse interviews with people living with HIV;

2. recruit, appoint and train two advisory group members to provide guidance on the overarching strategy and progress of the project;

3. build new relationships and strengthen existing connections with people living with HIV to facilitate future co-research through prioritising study findings and disseminating results;

4. up-skill and build capacity for qualitative research within a CBO.

## Methods and findings

Our approach builds on the reflections of two co-produced research projects [21, 22] and published recommendations [23, 24]. Due to the COVID-19 pandemic, the ‘community-based’ element of our project had to be transformed to work in an online/virtual space, rather than face-to-face. Here, we use the broad term ‘co-researchers’ to describe both the lived experience qualitative researchers and advisory group members, who were all living with HIV.

**Fig. 1:**
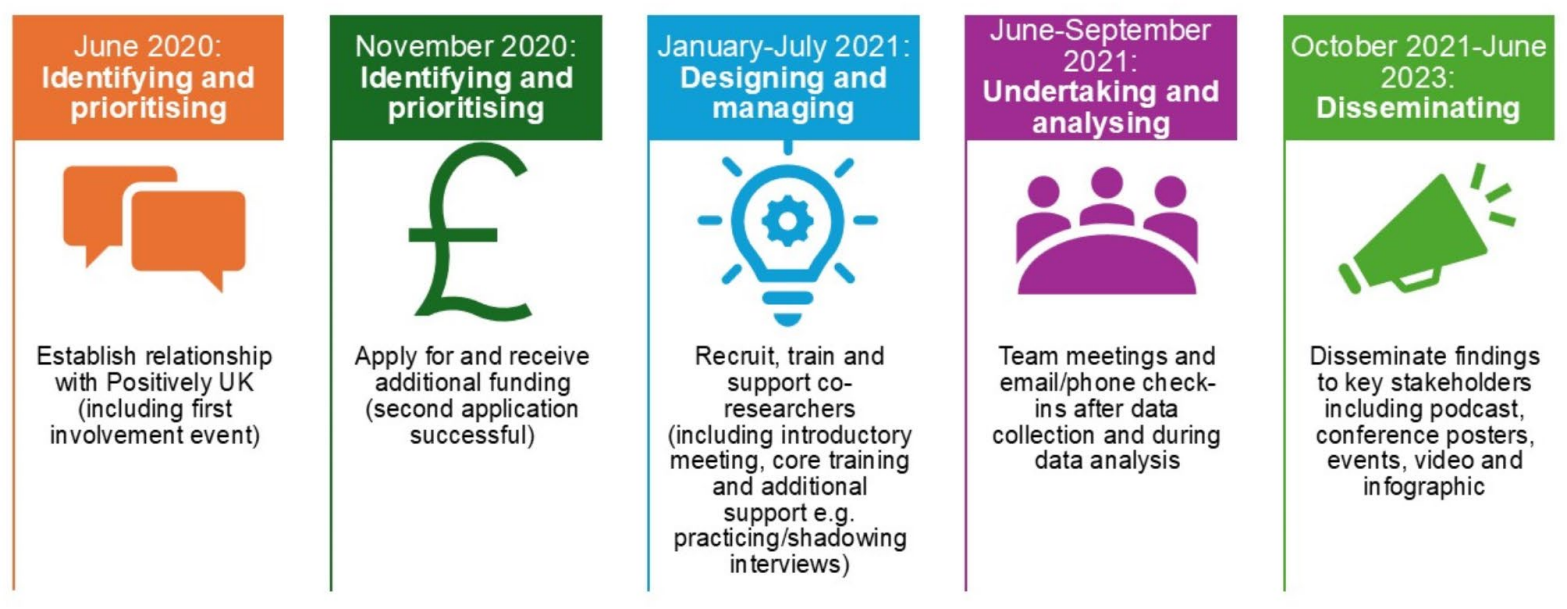
Project timeline overview (2020–2023) mapped to key stages in the research cycle

We frame our methods, reflections and recommendations using each stage of the research cycle according to the National Institute for Health and Care Research (NIHR) [25]. Figure 1 outlines our project timeline and Table 1 our recommendations for each stage of involvement in the research cycle.

**Table 1:** Summary of recommendations for each stage of research cycle

Stage of research cycle	Recommendations
Identifying and prioritising	• Academic researchers and public involvement practitioners should begin to build relationships with community-based organisations (CBOs) as early as possible. • Diversity and representation in the project team is essential to ensure all voices are heard. This includes sociodemographic factors such as age, gender, ethnicity and sexual orientation as well as years living with HIV (for people with lived experience involved). Conducting research remotely can also improve geographical representation within the team, particularly where people with lived experience live in rural or remote areas, although digital exclusion may be an issue. • Academic researchers and public involvement practitioners should identify any issues relating to technology/digital access, or knowledge, early on and ensure access or develop training to circumvent such issues arising. This may include offering additional avenues for those without access to technology to provide their insights to supplement online discussion groups.
Funding and commissioning	• Be flexible with funding budget – work with your community-based partner and adequately remunerate their involvement in the project, as well as the co-researchers. For example, understanding any equipment that might need to be ordered for your co-researchers (e.g. headphones) and preferences on the type of honorarium received (e.g. bank payment or vouchers). • Research funders and councils should develop more funding schemes to promote and support participatory approaches in research, with budgets ranging from low-level (advisory/consultation) to high-level (co-research) involvement.
Designing and managing	• Have an iterative and flexible approach to training that facilitates opportunities for co-researchers to provide feedback on the skills they feel most and least confident about. These insights can then be used to inform and enhance the design, content, and delivery of training sessions. • Training should be developed that is mindful of the histories, backgrounds, and experiences of the co-researchers (e.g. HIV-related stigma, traumatic experiences etc.). • Emotional well-being is a fundamental component of any type of participatory study and should be mandatory in training. • Links to support services (e.g. collaborating with a support organisation) can assist with developing appropriate tools and interventions. • Manage expectations on what can and cannot be achieved given resources and time. • Spend time getting to know the team on a personal level through icebreakers and allowing time in meetings for such personal information sharing discussions – what skills, hobbies and interests do people have? This can help co-researchers settle in, feel comfortable and familiarise with a project where they may be new to research and/or collaborating with the researcher. • Explore what co-researchers hope to get out of the project (e.g. future research opportunities, forms of recognition, further training or skills development for roles outside of academia).
Undertaking and analysing	• Encourage co-researchers to voluntarily share their backgrounds either at the start or end of the interview process with participants to help build rapport (if they feel comfortable). • Manage expectations of co-researchers about “no shows” or participants withdrawing from the interview process by explaining this during training and at check-in sessions during data collection. • Schedule debriefing meetings after interviews – an initial check-in email immediately afterwards followed by a call/team meeting for specific issues to allow time for co-researchers to reflect on any concerns. • Co-researchers could also mentor and support each other in team meetings by sharing reflections from their experiences. • Development of more in-depth, in-person training on qualitative data analysis to facilitate learning, support, and active participation. • Ensure adequate time is scheduled for data analysis and consider creative methods of coding when institutional tools (e.g. NVivo) may not be available for co-researchers.
Disseminating	• All key stakeholders should be involved in planning the dissemination of the research findings as this collaboration could assist with prioritising who, how and where findings should be shared/disseminated. • Creative forms of dissemination (which are co-produced) help to increase research impact, particularly if developed to be accessible in terms of language and style.
Implementing and evaluating feedback	• Determine the best approach and timepoints for self-reflection at the start of the project, as a group, with this formally agreed by all team members. • Be open to continuing dialogue beyond the research project to self-reflect as academic researchers and co-researchers and make changes for future co-produced research.

### Identifying and prioritising: Building relationships and managing expectations

In 2020, we began collaborating with the peer-led HIV support charity, Positively UK [26]. One of the academic researchers (JB) was a Trustee of Positively UK at the time and was aware of the organisation’s motivation to develop internal research capacity, particularly amongst volunteers. Therefore, we were able to build a strong connection from an existing trusting relationship between a member of the academic research team (JB) and the CEO of the charity (SP). Our first community involvement activity was an online event^1^, ‘COVID-19 Community Involvement: Let’s Talk About… HIV care’, and aimed to gather insights on the challenges and opportunities presented to HIV care during the pandemic. The event, hosted by PERC on Zoom, was attended by 25 people from across the UK including people living with HIV, and providing HIV services and working at HIV charities. The event consisted of a short presentation and breakout room discussions (five rooms) each facilitated by a member of Positively UK and a PERC staff member (as a note-taker). The session aimed to shape the design and focus of the qualitative study. Priorities and views shared during this online session directly fed into the development of this project, with those in attendance highlighting the need to further explore the impact of COVID-19 on those living with HIV and the services and care available and received. Furthermore, insights gained were later used for the development of interview questions for subsequent qualitative studies as well as to identify interested participants. Details on the approach and findings are published elsewhere [12].

Following the event, we ran a debriefing session with all the facilitators to identify key messaging for the insight report [12] as well as reflections on the involvement activity.

#### Reflections

During the debrief, we identified what worked well and areas of improvement (Table 2). We identified how trust and rapport when building relationships is essential, particularly when all interactions are remote and with novice HIV researchers. Engaging regularly, early in the project, and with respect have been identified as important metrics when working with people with lived experiences [28]. Additionally, issues relating to access to technology and data were raised as well as ensuring that the research team was inclusive and diverse in terms of age, gender, ethnicity and geographical location.

**Table 2:** Reflections from Zoom event by facilitators. Adapted from Papageorgiou et al. [12]

What worked well	What could be improved
Attendees felt open to share their experiences from both a clinical and personal perspective	Issues of digital exclusion – other ways to involve for people who do not use, are unfamiliar with or do not have access to, Zoom
Having *Positively UK* facilitating and being part of the community involvement activity and research process – sharing experiences helped foster an enabling environment for attendees to open up	Limited time which meant some discussions had to end quickly
The facilitators involved were from diverse backgrounds and being a team of predominantly women worked well	Gender imbalance (one male co-facilitator) may result in bias
Having a mixed group of participants including clinicians allowed for in-depth discussions on different areas of HIV care (NHS services, voluntary sector etc.)	There were some technical issues with a few participants sending emails after the event to explain that they could not re-join the call after breakout discussions

#### Recommendations

• Academic researchers and public involvement practitioners should begin to build relationships with CBOs as early as possible.

• Diversity and representation in the project team is essential to ensure all voices are heard. This includes sociodemographic factors such as age, gender, ethnicity and sexual orientation as well as years living with HIV (for people with lived experience involved). Conducting research remotely can also improve geographical representation within the team, particularly where people with lived experience live in rural or remote areas, although digital exclusion may be an issue.

• Academic researchers and public involvement practitioners should identify any issues relating to technology/digital access, or knowledge, early on and ensure access or develop training to circumvent such issues arising. This may include offering additional avenues for those without access to technology to provide their insights to supplement online discussion groups.

### Funding and commissioning: flexibility and seeking opportunities

Due to the nature of an ongoing outbreak, we had to swiftly identify available funding to enhance and support the project, particularly the remuneration of co-researchers in line with NIHR guidelines [29]. We (VP, JB, HW, HJ) co-developed a funding application^2^ with our collaborator (SP) and secured a budget to cover the costs of two co-researchers and two advisory group members as part of the project.^3^ We later re-applied to the same funder for further funding, from a different scheme, to support the dissemination of research findings.

#### Reflections

We identified few appropriate funding schemes to support co-research as there were no specific funds for HIV research.

#### Recommendations

• Be flexible with your funding budget – work with your community-based partner and adequately remunerate their involvement in the project, as well as co-researchers. For example, understanding any equipment that might need to be ordered for your co-researchers (e.g. headphones) and preferences on the type of honorarium received (e.g. bank payment or vouchers).

• Research funders and councils should develop more funding schemes to promote and support participatory approaches in research, with budgets ranging from low-level (advisory/consultation) to high-level (co-research) involvement.

### Designing and managing: reciprocal learning

Two lived experience qualitative researchers and three advisory group members including one collaborator also with lived experience, were recruited and trained between January and October 2021. We designed our research approach based on published reflections from colleagues [21] and amended an ethics application already in place for another qualitative study^4^ to incorporate the participatory component for this project. As a result, most of the study documents were already created; however, co-researchers were asked to review and provide feedback on all the documents including the protocol, interview guide, participant information sheet, a demographics survey and resource list. The results of this activity are outlined elsewhere [30].

**Fig. 2:**
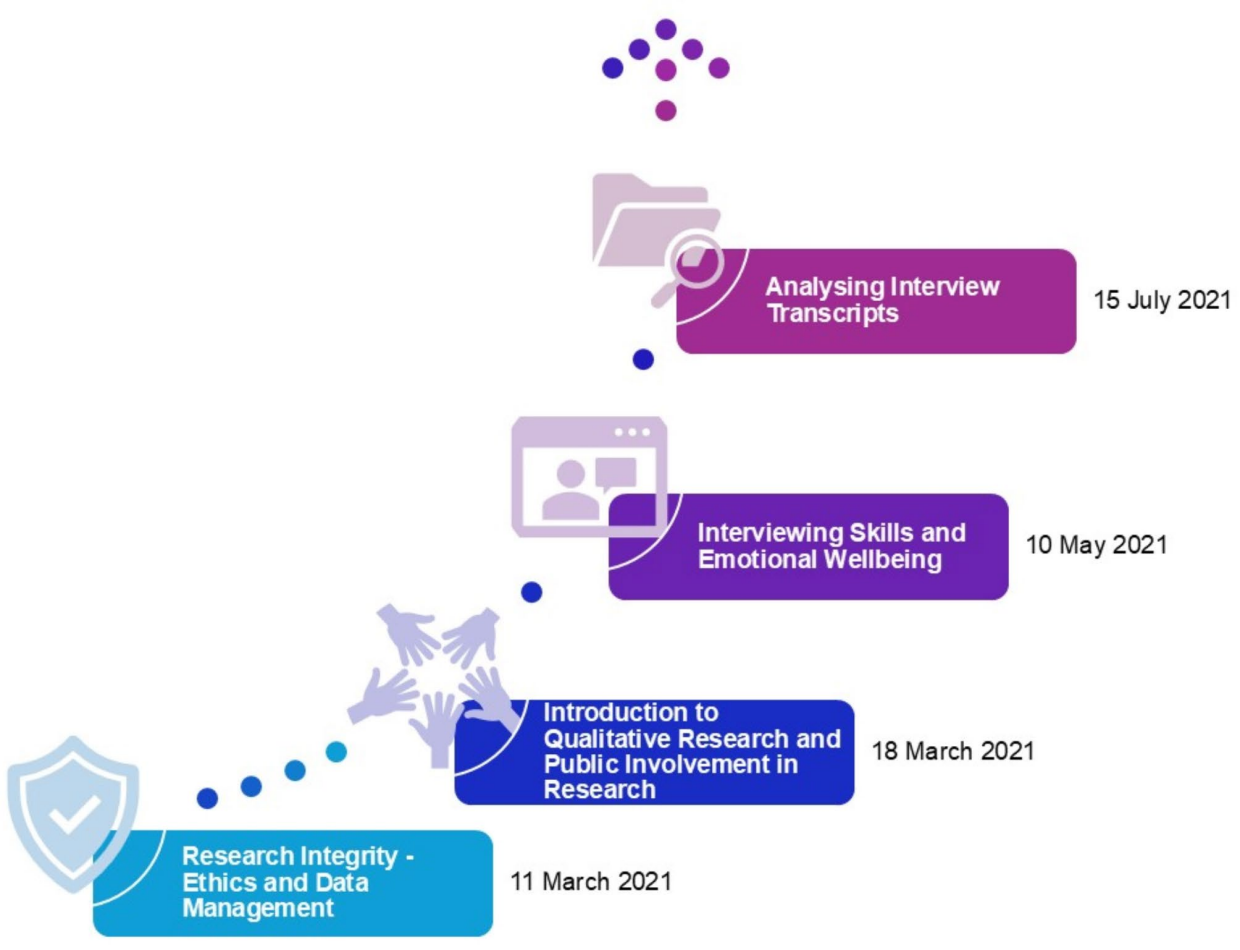
Overview of core co-researcher training session from March to July 2021

Core training sessions (Fig. 2) were designed and focused on building skills identified to be essential for roles from the research perspective as well as skills which were identified by co-researchers as areas of development using a “skills review” [31]. The skills review included reflections on experiences/knowledge of public involvement in medical research (e.g. contextual knowledge about HIV and COVID-19) and general skills, expertise, and knowledge (e.g. using virtual meeting platforms such as Zoom) [31]. We (VP, JB, HJ) used several existing resources to develop the training sessions. First, training on research integrity, including ethics, were designed in line with institutional requirements. As suggested by our collaborator at Positively UK (SP), training on ethics specifically focussed on issues around confidentiality, safeguarding and respecting boundaries. Ensuring appropriate support is in place for emotionally challenging research (for example, where team members have lived experience of the research topic) is essential and has also been reported in recent co-produced HIV studies as well as in other fields of health research [32, 33]. Second, the session on qualitative research and public involvement was adapted from Bees et al. [34]. Third, we developed our interviewing training to also cover emotional well-being of peer interviewers based on recommendations from other HIV researchers and organisations [23, 24, 35]. Finally, the training and approach of analysing interview transcripts was adapted from the DEPICT^5^ model [36]. Training session materials have been deposited online and are freely available to use [37].

Additionally, during some research team meetings and one-to-one discussions, we had brief training sessions on other aspects which were not previously covered. For example, we discussed how to conduct interviews using Zoom and how to present research in academic writing (specifically writing conference abstracts). We also used time for reflections on skills development during the project.

#### Reflections

Research team meetings provided time to reflect on all progress made and for learning and information sharing between researchers and people living with HIV (in both directions). One co-researcher explained how they appreciated the depth and breadth of training,

…the training is really helpful; you get to realise the amount of work and people that are involved in a research project.

#### Recommendations

• Have an iterative and flexible approach to training that facilitates opportunities for co-researchers to provide feedback on the skills they feel most and least confident about. These insights can then be used to inform and enhance the design, content, and delivery of training sessions.

• Training should be developed that is mindful of the histories, backgrounds, and experiences of the co-researchers (e.g., HIV-related stigma, traumatic experiences etc.).

• Emotional well-being is a fundamental component of any type of participatory study and should be mandatory in training.

• Links to support services (e.g. collaborating with a support organisation) can assist with developing appropriate tools and interventions.

• Manage expectations on what can and cannot be achieved given resources and time.

• Spend time getting to know the team on a personal level through icebreakers and allowing time in meetings for such personal information sharing discussions – what skills, hobbies and interests do people have? This can help co-researchers settle in, feel comfortable and familiarise with a project where they may be new to research and/or collaborating with the researcher.

• Explore what co-researchers hope to get out of the project. For example, future research opportunities, forms of recognition, further training, or skills development for roles (outside of academia).

### Undertaking and analysing: flexibility and ensuring all voices are heard

We (VP) sampled participants from the 2017 Positive Voices study which is a nationally representative survey on the health and well-being of people living with HIV in England and Wales [38]. We approached 260 of 1,231 participants with valid contact details who were happy to be contacted about future research. Our recruitment approach and methods are outlined in full elsewhere [30]. In total, 19 interviews were conducted with people living with HIV between June and August 2021; five of these interviews were undertaken by two co-researchers and 14 by two academic researchers.

We scheduled check-in meetings with co-researchers and research team meetings for all members on Zoom or by phone. Check-in meetings were scheduled immediately after any interviews to discuss any issues raised as well as time scheduled during fortnightly team meetings to discuss any difficult conversations. Our partner, Positively UK, were also available to provide peer support, if required by co-researchers. Both qualitative co-researchers had a criminal record check by the Disclosure Barring Service (DBS) before conducting any interviews.

Google Jamboard was the preferred method for recording notes from research team meetings. For the qualitative analysis, each team was assigned a minimum of one transcript to code by thematic analysis, with co-researchers given transcripts from interviews they completed. All co-researchers received training on the importance of data protection and management [37] and had signed a non-disclosure agreement prior to data collection which outlined the importance of handling any sensitive data (i.e. transcripts). Transcripts were password-protected before transfer to an encrypted Microsoft OneDrive for Business folder with permissions set for ‘view only’ to prevent downloads of the file to any personal devices. We completed a whole team analysis using Google Jamboard to manage codes, categories, themes and sub-themes. All the data (code, categories, themes) were then transferred into NVivo as a coding framework, with coding applied to all transcripts by one of the academic researchers (VP).

#### Reflections

Some tasks were easier to co-produce than others; for example, most co-researchers were confident when interviewing if this was framed as a conversation and they were able to follow a topic guide with open-ended prompts or examples given as prompts. However, data analysis using a participatory approach was more challenging as the interview transcripts were lengthy given that the interviews lasted up to 90 minutes. Moreover, the analysis also took place during the summer months when the team members were on leave which delayed the process. Additionally, some team members experienced technical issues accessing the transcripts. These were troubleshooted by the lead researcher, who in some instances had to share the correct password for members to open files, by phone. Involving qualitative co-researchers may have improved the experiences of participants by helping to build deeper rapport, free from judgement and from a place of understanding that enhances knowledge creation although, the approach may also introduce an emotional burden or blur personal and professional boundaries [39, 40].

#### Recommendations

• Encourage co-researchers to voluntarily share their backgrounds either at the start or end of the interview process with participants to help build rapport (if they feel comfortable).

• Manage expectations of co-researchers about “no shows,” or participants withdrawing from the interview process by explaining this during training and at check-in sessions during data collection.

• Schedule debriefing meetings after interviews – an initial check-in email immediately afterwards followed by a call/team meeting for specific issues to allow time for co-researchers to reflect on any concerns.

• Co-researchers could also mentor and support each other in team meetings by sharing reflections from their experiences.

• Development of more in-depth, in-person training on qualitative data analysis to facilitate learning, support, and active participation.

• Ensure adequate time is scheduled for data analysis and consider creative methods of coding when institutional tools (e.g. NVivo) may not be available for co-researchers.

### Disseminating: impact beyond academic outputs

**Fig. 3:**
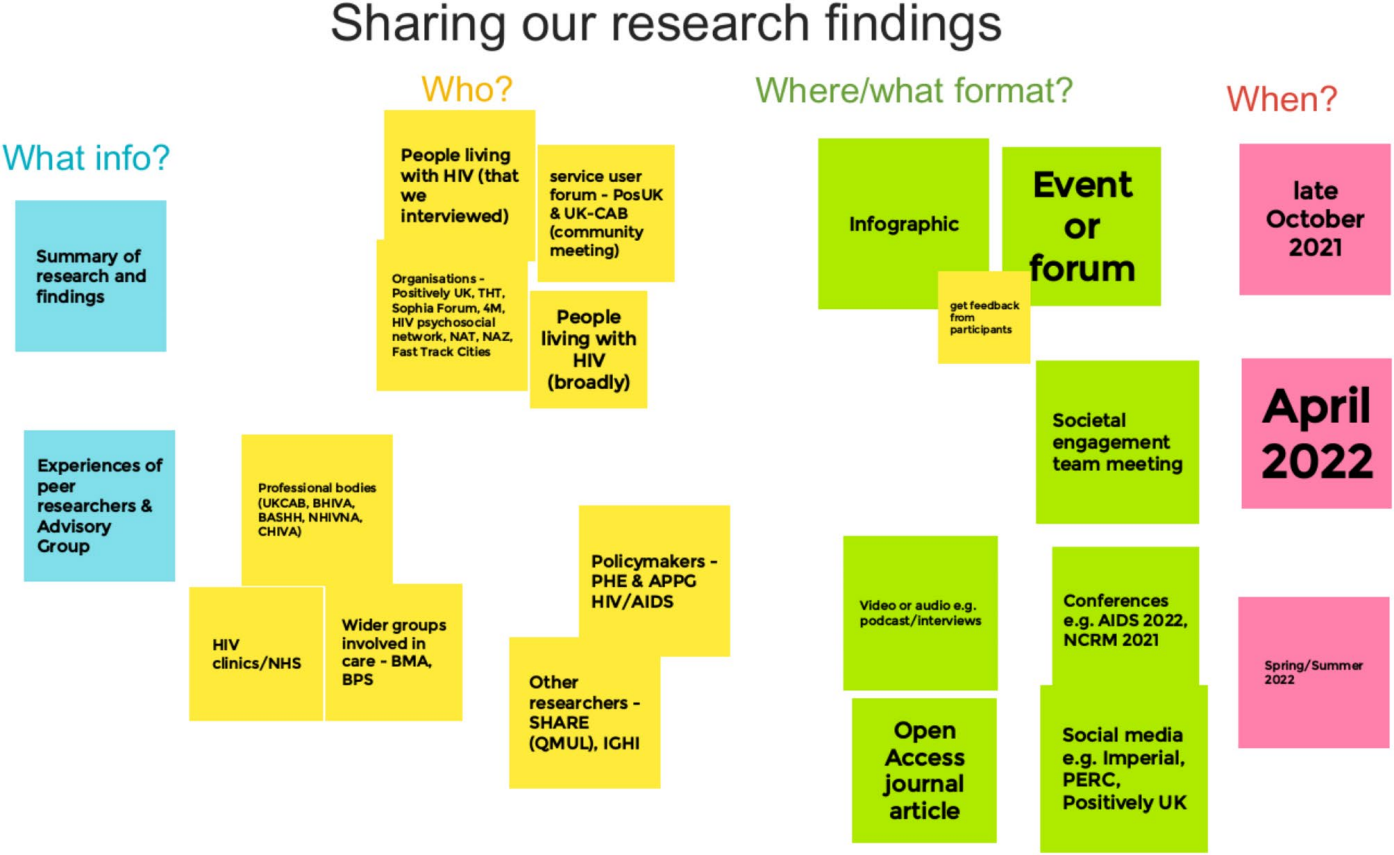
Dissemination plan co-designed by the group using Google Jamboard. Taken from Papageorgiou (2022) [30]

We worked as a group to develop a dissemination plan (Fig. 3) and prioritised outputs according to stakeholders and timings; for example, community and participant-facing communications were prioritised in line with ‘best practice’ to recognise and appropriately respect their contributions to the research [41, 42].

Our outputs include:

1. A co-produced reflective workshop at a conference alongside other participatory projects on “building and sustaining partnerships” co-designed and co-facilitated by co-researchers (HH, WT) [33, 43].

2. An infographic presenting our preliminary findings for all study participants.

3. A podcast episode on our experiences working together during the pandemic featuring reflections from an academic researcher (VP) and co-researcher (HH) [44].

4. A co-produced workshop for academic staff/students on how to conduct co-research co-facilitated by a co-researcher (WT).

5. A co-produced showcase event to highlight the research findings and conduct participatory activities with key stakeholders, including prioritisation of research findings [45, 46]. This included the development of a short video on our experiences working together and why co-research promotes more meaningful and impactful research [47] and a visual illustration was produced during the event [46]. Key stakeholders invited to attend the event included people living with HIV (e.g. those interviewed as part of the study), HIV charities and CBOs, policymakers, pharmaceutical companies, professional medical bodies, researchers and clinicians.

6. Two co-designed conference posters at international conferences; one was presented by a co-researcher (HH) [48, 49]. One co-researcher (WT) also received a scholarship to attend a conference virtually.

7. A blog published on the Positively UK website [50] providing a summary of our project and signposting to further information on our study webpage [51].

#### Reflections

Co-researchers wanted any public-facing documents to be prioritised and were keen for these to be visually and audibly accessible, rather than in word format); these outputs also had the greatest reach [30]. Key stakeholders from the showcase event assisted us to disseminate our findings and prioritising the importance of each finding [30, 46].

#### Recommendations

• All key stakeholders should be involved in planning the dissemination of the research findings as this collaboration could assist with prioritising who, how and where findings should be shared/disseminated.

• Creative forms of dissemination (which are co-produced) help to increase research impact, particularly if developed to be accessible in terms of language and style.

### Implementing and evaluating impact: embedding continuous reflection

Initially, this step was to be embedded throughout the project through guided self-reflection activities. However, time constraints and limited uptake from co-researchers, resulted in this occurring at the end of the project and in more informal ways. For example, feedback from co-researchers received by emails and phone were recorded and published elsewhere [30].

#### Reflections

We aimed to advocate for empowerment as a group by ensuring that all voices were heard, actions were taken, and the impact of involvement was recorded. However, the uncertainty of the pandemic meant that we had to be flexible and open to unexpected challenges including life events and the nuances of conducting research during a pandemic.

Several of the co-researchers have continued to be involved in research (JA, HH, WT) and WT has since developed and led her own research project which aligns with literature on empowerment [6, 52]. Reflections of involvement have continued beyond the closure of this distinct research project.

#### Recommendations

• Determine the best approach and timepoints for self-reflection at the start of the project, as a group, with this formally agreed by all team members.

• Be open to continuing dialogue beyond the research project to self-reflect as academic researchers and co-researchers and make changes for future co-produced research.

## Conclusions

HIV research lends itself to co-research due to its underlying roots in advocacy and empowerment. However, the COVID-19 pandemic raised specific challenges to conducting co-research which required iterative adaptations and creativity. Well-designed and thorough training sessions are key to ensuring co-researchers feel confident and supported when joining a research team. Creative forms of dissemination which are co-designed and co-produced by people with lived experience enhance the reach and impact of research projects, ultimately driving transformation at a service level. Developing trust between research team members early on can enhance the experiences of all involved and facilitate the development of confidence, new skills, empowerment and relationships throughout the project. Overall, our co-produced study provided the opportunity for the experiential knowledge of team members to influence the approach and interpretation of findings. This ensured that the research remained meaningful and relevant to people with HIV and the findings and recommendations were grounded in lived experience. Our approach resulted in the enhancement of research skills and capabilities within the HIV community and has led to co-researchers continuing to be involved with, or beginning to lead their own, research.

## Data Availability

No datasets were generated or analysed during the current study.

## Footnotes

1 This built on the NIHR Imperial BRC Patient Experience Research Centre’s (PERC) new model of community involvement events (‘COVID-19 Community Involvement: Let’s Talk About…’) to gather rapid insights on various topics during the outbreak [27] which was informed by early community involvement [20].

2 This was our second attempt securing this seed funding. We used the feedback received from our first, unsuccessful application to strengthen our second application.

3 We subsequently applied for additional funding to recruit two advisory group members following the high-quality applications from the initial round of recruitment.

4 The overarching study was not undertaken by the research group.

5 The DEPICT model includes: **D**ynamic reading, Engaged codebook development, Inclusive reviewing and summarising of categories, **C**ollaborative analysing and **T**ranslating [36].

## Abbreviations

CBPR: Community-Based Participatory Research
CBO: Community-Based Organisation
HIV: Human immunodeficiency virus
GIPA: Greater Involvement of People Living with HIV/AIDS
MIPA: Meaningful Involvement of People Living with HIV/AIDS
NIHR: National Institute for Health and Care Research
PERC: (NIHR Imperial Biomedical Research Centre) Patient Experience Research Centre

## References

[1] National Institute for Health and Care Research. Glossary [Internet]. 2022. Available from: https://www.nihr.ac.uk/glossary/

[2] Hickey G, Brearley S, Coldham T, Denegri S, Green G, Staniszewska S et al. Guidance on co-producing a research project [Internet]. Southampton; 2018. Available from: https://www.learningforinvolvement.org.uk/wp-content/uploads/2021/04/Guidance-on-co-producing-a-research-project-2022.pdf

[3] Blumenthal DS. Is Community-Based participatory research possible? Am J Prev Med. 2011;40:386–9. 21335275 10.1016/j.amepre.2010.11.011 PMC3976961

[4] Messiha K, Chinapaw MJM, Ket HCFF, An Q, Anand-Kumar V, Longworth GR, et al. Systematic review of contemporary theories used for Co-creation, Co-design and Co-production in public health. J Public Health. 2023;45:723–37. 10.1093/pubmed/fdad046 PMC10470345 37147918

[5] Perkins DD, Zimmerman MA. Empowerment theory, research, and application. Am J Comm Psychol. 1995;23:569–79. 10.1007/BF02506982 8851340

[6] Gupta V, Eames C, Golding L, Greenhill B, Qi R, Allan S, et al. Understanding the identity of lived experience researchers and providers: a conceptual framework and systematic narrative review. Res Involv Engagem. 2023;9:26. 37095587 10.1186/s40900-023-00439-0 PMC10127294

[7] Hutchinson A, Lovell A. Participatory action research: moving beyond the mental health ‘service user’ identity. Psychiatric Ment Health Nurs. 2013;20:641–9. 10.1111/jpm.12001 23167824

[8] Charlton JI. Nothing about Us without Us: disability oppression and empowerment. Univ of California; 1998.

[9] AIDS United, US People Living with HIV Caucus. Embodying Meaningful Involvement of People Living with HIV: History and Lessons Learned from the Community [Internet]. Washington, DC. 2019 Sep. Available from: http://aidsunitedbtc.wpengine.com/wp-content/uploads/2021/05/MIPA_Toolkit_FINAL.pdf

[10] Brizay U, Golob L, Globerman J, Gogolishvili D, Bird M, Rios-Ellis B, et al. Community-academic partnerships in HIV-related research: a systematic literature review of theory and practice. J Int AIDS Soc. 2015;18:19354. 25630823 10.7448/IAS.18.1.19354 PMC4309828

[11] Jiang H, Zhou Y, Tang W. Maintaining HIV care during the COVID-19 pandemic. Lancet HIV. 2020;7:e308–9. 32272084 10.1016/S2352-3018(20)30105-3 PMC7239666

[12] Papageorgiou V, Cooper E, Bruton J, Petretti S, Pristerà P, Ward H, Insight Report. COVID-19 Community Involvement - Let’s Talk About… HIV Care [Internet]. Patient Experience Research Centre, Imperial College London (in collaboration with Positively UK); 2020 Jul. Available from: 10.25561/93710

[13] Lesko CR, Bengtson AM. HIV and COVID-19: intersecting epidemics with many unknowns. Am J Epidemiol. 2021;190:10–6. 32696057 10.1093/aje/kwaa158 PMC7454306

[14] Waterfield KC, Shah GH, Etheredge GD, Ikhile O. Consequences of COVID-19 crisis for persons with HIV: the impact of social determinants of health. BMC Public Health. 2021;21:299. 33546659 10.1186/s12889-021-10296-9 PMC7863613

[15] Piercy H, Kelly S, Wills M, Croston M. Psychological impact of caring during the COVID-19 pandemic on HIV nurses. Br J Nurs. 2022;31:S10–5. 35019747 10.12968/bjon.2022.31.1.S10

[16] Pantelic M, Martin K, Fitzpatrick C, Nixon E, Tweed M, Spice W, et al. I have the strength to get through this using my past experiences with HIV: findings from a mixed-method survey of health outcomes, service accessibility, and psychosocial wellbeing among people living with HIV during the Covid-19 pandemic. AIDS Care. 2022;34:821–7. 34530649 10.1080/09540121.2021.1975628

[17] British HIV Association and Terrence Higgins Trust (THT). Comment from BHIVA and THT on UK Government Guidance on Coronavirus (COVID-19), Social Distancing to Protect Vulnerable Adults and Shielding to Protect Extremely Vulnerable Adults [Internet]. 2020. Available from: https://www.bhiva.org/comment-from-BHIVA-and-THT-on-UK-Government-guidance-on-Coronavirus-COVID-19

[18] British HIV Association. COVID-19 & shielding: advice for HIV clinicians, GPs and people living with HIV [Internet]. 2020. Available from: https://www.bhiva.org/COVID-19-and-shielding-advice-for-HIV-clinicians-GPs-and-people-living-with-HIV

[19] British HIV Association. Shielding text messages sent in error to people living with HIV, response from the British HIV Association (BHIVA) and the Terrence Higgins Trust (THT) [Internet]. 2020 [cited 2020 May 11]. Available from: https://www.bhiva.org/shielding-text-messages-sent-in-error-to-people-living-with-HIV

[20] Pristerà P, Papageorgiou V, Kaur M, Atchison C, Redd R, Bowman L et al. Report 14: Online community involvement in COVID-19 research & outbreak response: early insights from a UK perspective [Internet]. Imperial College London; 2020 Apr. Available from: 10.25561/77842

[21] Dewa LH, Lawrence-Jones A, Crandell C, Jaques J, Pickles K, Lavelle M, et al. Reflections, impact and recommendations of a co-produced qualitative study with young people who have experience of mental health difficulties. Health Expect. 2020;24:134–46. 32515538 10.1111/hex.13088 PMC8137486

[22] Cook L, Rothstein P, Emeh L, Frumiento P, Kennedy D, McNicholas D, et al. In the physical to digital transition with friends—A story of performing inclusive research together no matter what life throws at you. Brit J Learn Disabil. 2021;49:271–81. 34566467 10.1111/bld.12408 PMC7611723

[23] Ibáñez-Carrasco F, Watson JR, Tavares J. Supporting peer researchers: recommendations from our lived experience/expertise in community-based research in Canada. Harm Reduct J. 2019;16:55. 31481067 10.1186/s12954-019-0322-6 PMC6724244

[24] Kaida A, Carter A, Nicholson V, Lemay J, O’Brien N, Greene S, et al. Hiring, training, and supporting peer research associates: operationalizing community-based research principles within epidemiological studies by, with, and for women living with HIV. Harm Reduct J. 2019;16:47. 31319894 10.1186/s12954-019-0309-3 PMC6637632

[25] National Institute for Health and Care Excellence. Briefing notes for researchers - public involvement in NHS, health and social care research [Internet]. 2021 Apr. Available from: https://www.nihr.ac.uk/documents/briefing-notes-for-researchers-public-involvement-in-nhs-health-and-social-care-research/27371

[26] Positively UK [Internet]. 2019 [cited 2021 Nov 23]. Available from: https://positivelyuk.org/

[27] Patient Experience Research Centre. COVID-19 Community Involvement [Internet]. Imperial College London. [cited 2022 Nov 2]. Available from: https://www.imperial.ac.uk/medicine/research-and-impact/groups/patient-experience-research-centre/covid-19/covid19communityinvolvement/

[28] Riches L, Ridgway L, Edwards L. Co-learning commentary: a patient partner perspective in mental health care research. Res Involv Engagem. 2023;9:24. 37072880 10.1186/s40900-023-00435-4 PMC10114418

[29] NIHR Centre for Engagement and Dissemination. Recognition payments for public contributors (Version 1.1 - July 2020) [Internet]. 2020. Available from: https://www.nihr.ac.uk/documents/centre-for-engagement-and-dissemination-recognition-payments-for-public-contributors/24979

[30] Papageorgiou V, HIV. COVID-19 and health and well-being: a mixed-methods exploration of the impact of structural and social determinants [Internet]. Imperial College London; 2022 [cited 2023 Nov 17]. Available from: 10.25560/107581

[31] Papageorgiou V, Bruton P, Johnson H, Ward H. Supporting material for co-researchers [Internet]. 2022 Nov. Available from: 10.25561/100339

[32] Kasadha B, Tariq S, Namiba A, Freeman-Romilly N, Moepi N, Letting G, et al. Hearing the silence and silenced: Co‐Producing research on Infant‐Feeding experiences and practices with black women with HIV. Sociol Health Illn. 2025;47:e13871. 39743738 10.1111/1467-9566.13871 PMC11693977

[33] Papageorgiou V, Dewa LH, Bruton J, Murray K-K, Hewlett N, Thamm W, et al. Building bridges’: reflections and recommendations for co-producing health research. Res Involv Engagem. 2023;9:113. 38057931 10.1186/s40900-023-00528-0 PMC10702073

[34] Bee P, Brooks H, Callaghan P, Lovell K, editors. A research handbook for patient and public involvement researchers. Manchester University; 2018.

[35] AIDS Bereavement and Resiliency Program of Ontario. Essential Tools for Support and Stability: Worker Resource Kit [Internet]. 2016 May. Available from: https://abrpo.org/resources/essential-tools-for-support-and-stabiliity/

[36] Flicker S, Nixon SA. The DEPICT model for participatory qualitative health promotion research analysis piloted in Canada, Zambia and South Africa. Health Promot Int. 2015;30:616–24. 24418997 10.1093/heapro/dat093 PMC4542917

[37] Papageorgiou V, Bruton J, Johnson H, Ward H. Peer Research Training Resource [Internet]. Patient Experience Research Centre, Imperial College London; 2022. Available from: 10.25561/94819

[38] Kall M, Kelly C, Auzenbergs M, Delpech V. Positive voices: the National survey of people living with HIV - findings from the 2017 survey. London: Public Health England; 2020 Jan.

[39] Devotta K, Woodhall-Melnik J, Pedersen C, Wendaferew A, Dowbor TP, Guilcher SJ, et al. Enriching qualitative research by engaging peer interviewers: a case study. Qualitative Res. 2016;16:661–80.

[40] Jørgensen CR, Eskildsen NB, Thomsen TG, Nielsen ID, Johnsen AT. The impact of using peer interviewers in a study of patient empowerment amongst people in cancer follow-up. Health Expect. 2018;21:620–7. 29206313 10.1111/hex.12655 PMC5980595

[41] Health Research Authority. Publication and dissemination of research findings [Internet]. 2021. Available from: https://www.hra.nhs.uk/planning-and-improving-research/best-practice/publication-and-dissemination-research-findings/

[42] Medical Research Council. Framework on the feedback of health-related findings in research. Available from: https://www.ukri.org/publications/framework-on-the-feedback-of-health-related-findings-in-research/

[43] NIHR Applied Research Collaboration Northwest London. Co-researchers drive co-production workshop [Internet]. 2021 [cited 2022 Nov 4]. Available from: https://www.arc-nwl.nihr.ac.uk/news/co-researchers-drive-co-production-workshop

[44] Pozcast. Peer Research across the pond: a fascinating UK study exposes barriers and bias bolstered by the pandemic [Internet]. [cited 2022 May 4]. Available from: https://www.positiveeffect.org/pozcast/se2ep15-peer-research-across-the-pond-a-fascinating-uk-study-exposes-barriers-and-bias-bolstered-by-the-pandemic

[45] Johnson H.HIV, Co-Production. Showcase: Sharing learnings from a participatory research project (Part 1) [Internet]. Patient Experience Research Centre. 2022 [cited 2022 Aug 23]. Available from: https://blogs.imperial.ac.uk/perc/2022/07/06/hiv-co-production-showcase-sharing-learnings-from-a-participatory-research-project-part-1/

[46] Johnson H. HIV, Co-Production. Showcase: Sharing learnings from a participatory research project (Part 2) [Internet]. Patient Experience Research Centre. 2022 [cited 2022 Aug 23]. Available from: https://blogs.imperial.ac.uk/perc/2022/08/05/hiv-co-production-showcase-sharing-learnings-from-a-participatory-research-project-part-2/

[47] Imperial Medicine. Why Co-Production? Reflections from an HIV Research Study [Internet]. 2022 [cited 2022 Oct 6]. Available from: https://www.youtube.com/watch?v=MvyKA3k5J9o

[48] Papageorgiou V, Bruton J, Dsouza K, Hamza H, Thamm W, Anderson J, et al. Experiences of the COVID-19 epidemic: a participatory qualitative study with people living and/or working with HIV in the UK. Canada: Montreal; 2022.

[49] Papageorgiou V, Hamza H, Anderson J, Bruton J, Dsouza K, Johnson H, et al. Co-production in HIV research: reflections from a study on Building relationships, conducting qualitative research and developing skills remotely. Stockholm; 2023.

[50] Positively UK. Research as co-production: impact of COVID-19 on people living with HIV in the UK [Internet]. 2022 [cited 2025 Mar 27]. Available from: https://positivelyuk.org/research-as-co-production-impact-of-covid-19-on-people-living-with-hiv-in-the-uk/

[51] Patient Experience Research Centre. Impact of COVID-19 on people living with HIV in the UK [Internet]. 2022 [cited 2025 Mar 27]. Available from: https://www.imperial.ac.uk/patient-experience-research-centre/covid-19/covid-19-research/hiv-care/

[52] Jones M, Pietilä I. Personal perspectives on patient and public involvement – stories about becoming and being an expert by experience. Sociol Health Illn. 2020;42:809–24. 32072657 10.1111/1467-9566.13064

